# Factors underlying masking release by voice-gender differences and spatial separation cues in multi-talker listening environments in listeners with and without hearing loss

**DOI:** 10.3389/fnins.2022.1059639

**Published:** 2022-11-23

**Authors:** Yonghee Oh, Curtis L. Hartling, Nirmal Kumar Srinivasan, Anna C. Diedesch, Frederick J. Gallun, Lina A. J. Reiss

**Affiliations:** ^1^Department of Otolaryngology and Communicative Disorders, University of Louisville, Louisville, KY, United States; ^2^National Center for Rehabilitative Auditory Research, VA Portland Health Care System, Portland, OR, United States; ^3^Department of Otolaryngology, Oregon Health & Science University, Portland, OR, United States; ^4^Department of Speech-Language Pathology & Audiology, Towson University, Towson, MD, United States; ^5^Department of Communication Sciences and Disorders, Western Washington University, Bellingham, WA, United States

**Keywords:** voice-gender release from masking, spatial release from masking, binaural pitch fusion, localization acuity, hearing loss, hearing aid (HA)

## Abstract

Voice-gender differences and spatial separation are important cues for auditory object segregation. The goal of this study was to investigate the relationship of voice-gender difference benefit to the breadth of binaural pitch fusion, the perceptual integration of dichotic stimuli that evoke different pitches across ears, and the relationship of spatial separation benefit to localization acuity, the ability to identify the direction of a sound source. Twelve bilateral hearing aid (HA) users (age from 30 to 75 years) and eleven normal hearing (NH) listeners (age from 36 to 67 years) were tested in the following three experiments. First, speech-on-speech masking performance was measured as the threshold target-to-masker ratio (TMR) needed to understand a target talker in the presence of either same- or different-gender masker talkers. These target-masker gender combinations were tested with two spatial configurations (maskers co-located or 60° symmetrically spatially separated from the target) in both monaural and binaural listening conditions. Second, binaural pitch fusion range measurements were conducted using harmonic tone complexes around a 200-Hz fundamental frequency. Third, absolute localization acuity was measured using broadband (125–8000 Hz) noise and one-third octave noise bands centered at 500 and 3000 Hz. Voice-gender differences between target and maskers improved TMR thresholds for both listener groups in the binaural condition as well as both monaural (left ear and right ear) conditions, with greater benefit in co-located than spatially separated conditions. Voice-gender difference benefit was correlated with the breadth of binaural pitch fusion in the binaural condition, but not the monaural conditions, ruling out a role of monaural abilities in the relationship between binaural fusion and voice-gender difference benefits. Spatial separation benefit was not significantly correlated with absolute localization acuity. In addition, greater spatial separation benefit was observed in NH listeners than in bilateral HA users, indicating a decreased ability of HA users to benefit from spatial release from masking (SRM). These findings suggest that sharp binaural pitch fusion may be important for maximal speech perception in multi-talker environments for both NH listeners and bilateral HA users.

## Introduction

Multi-talker listening environments occur when multiple talkers with various voice characteristics and spatial locations interact with each other. Those multi-talker listening situations present a challenging auditory environment which can make the task of target speech perception remarkably difficult for listeners due to masking effects created by the abundance of interfering background talkers (maskers). This situation is often referred to as the “cocktail party” phenomenon (Cherry, 1953).

Many previous studies have reported that there are two major acoustic cues that can improve speech segregation performance of a target message in listening environments like the “cocktail party” (Brungart, 2001; Albogast et al., 2002; Darwin et al., 2003; Ericson et al., 2004; Allen et al., 2008; Brungart et al., 2009; Best et al., 2011; Litovsky, 2012; Gallun et al., 2013; Srinivasan et al., 2016; Gaudrain and Başkent, 2018; Oh et al., 2021). One of these acoustic cues is vocal-characteristic differences between target and maskers that are a result of differences in talker gender (e.g., fundamental frequency differences, vocal-tract length differences, etc.) and the other is spatial separation between target and maskers (e.g., co-located vs. spatially separated talkers). Here, the improvement they can provide for speech segregation is referred to as “release from masking.” Specifically, the release from masking by the cues from vocal-characteristic differences is termed “voice-gender release from masking” (VGRM), and the masking release by spatial separation cues is termed “spatial release from masking” (SRM). It should be noted that the term VGRM was originally proposed in the study by Oh and Reiss, 2017a,b and used in their other studies (Oh et al., 2021, 2022). Here, “gender” denotes the classical categorization of a talker’s voice with their assigned sex at birth. Different terms have been used in previous speech-on-speech masking studies (e.g., “sex-mismatch benefits” Richter et al., 2021).

Previous studies have explored VGRM in isolation and have found that differences in voice characteristics between talkers of different genders lead to greater masking release than the differences in voice characteristics between talkers of the same gender for normal hearing (NH) listeners (Brungart, 2001; Ericson et al., 2004; Brungart et al., 2009). Studies have also explored SRM in isolation and have established that NH listeners benefit significantly from spatial separation cues between the target and competing maskers, beginning at separations as small as 2°, and that SRM benefit generally improves with increasing degrees of separation (Allen et al., 2008; Best et al., 2011; Litovsky, 2012; Gallun et al., 2013; Srinivasan et al., 2016; Yost, 2017).

While these findings are important, few studies have explored the interaction between these two cues together and their influences on SRM and VGRM. One recent study by Oh et al. (2021) found that there is an unequal perceptual weighting between the VGRM and SRM that NH listeners achieve across a spatial field. That is, at smaller spatial separations (up to 15–30°) between target and maskers, VGRM is more dominant than SRM, and at larger separations, (greater than 30 up to 60°) the perceptual weighting is reversed and SRM is more dominant than VGRM. Additionally, there was a clear point of intersection between this reversal of VGRM and SRM dominance where the magnitude of masking release for SRM and VGRM was equal.

In hearing-impaired (HI) listeners, bilateral device use including hearing aid (HA) and/or cochlear implant (CI) can be a major factor for binaural listening advantages in both voice-gender difference and spatial separation cues (Litovsky et al., 2006; Marrone et al., 2008; Visram et al., 2012; Bernstein et al., 2016). However, benefits from bilateral devices are highly variable, and often provide little speech perception benefit or even interfere with speech perception, compared to monaural device use (Litovsky et al., 2006; Ching et al., 2007; Reiss et al., 2016; Reiss and Molis, 2021). Reduced benefits of voice-gender differences in HI listeners could be attributed to poorer monaural frequency resolution for representation of pitch or even vocal tract length cues for voice pitch discrimination. Alternatively, recent findings suggest that reduced benefits from voice-gender difference could be explained by an increased likelihood to integrate dichotic stimuli that evoke different pitches between two ears into a single fused sound, which is termed binaural pitch fusion (Reiss and Molis, 2021; Oh et al., 2022). Generally, binaural pitch fusion is narrow in NH listeners because the two ears provide essentially matched spectral information for a given signal. In contrast, HI listeners can exhibit abnormally broad binaural pitch fusion, i.e., can fuse stimuli with pitches differing by up to 3–4 octaves across ears into a single percept (Reiss et al., 2014, 2017, 2018a,b; Oh and Reiss, 2017b, 2020). Thus, broad binaural pitch fusion appears to be detrimental, and could negatively impact the ability to segregate out multiple voices of different pitches in complex environments. In the current study, as the first goal, we investigated whether variability in binaural pitch fusion may explain some of the variability in voice-gender difference benefits in a common speech-on-speech masking task similar to those used in the previous studies.

Similarly, reduced benefits of spatial separation have previously been attributed to aging, hearing loss, poor sound source localization abilities, and a combination of those factors (Gallun et al., 2005, 2013; Best et al., 2011; Gifford et al., 2014; Füllgrabe et al., 2015; Srinivasan et al., 2016, 2021; Swaminathan et al., 2016; Ellinger et al., 2017; Baltzell et al., 2020). Their studies found aging and hearing loss could contribute to the reduction in SRM interdependently or independently (Gallun et al., 2013; Srinivasan et al., 2016). In addition, reduced temporal and spectral processing caused by either aging or hearing loss could reduce the ability to use spatial cues to segregate different auditory streams (Best et al., 2011; Füllgrabe et al., 2015; Srinivasan et al., 2016). There has also been some evidence showing that absolute sound localization ability from the processing of interaural time differences (ITDs) and interaural level differences (ILDs) could contribute to SRM (Gallun et al., 2005; Gifford et al., 2014; Srinivasan et al., 2016; Swaminathan et al., 2016; Ellinger et al., 2017; Baltzell et al., 2020). Most of their studies argued that the limited access to those localization cues could be explained by the interaction between aging and hearing loss. In the current study, as the second goal, we investigated whether variability in listener’s absolute sound localization ability may explain some of the variability in SRM in speech-on-speech masking.

The overall goal of this study was to measure two different types of masking releases due to (1) the voice-gender differences between talkers (i.e., VGRM); and (2) the spatial separation between talkers (i.e., SRM), and investigate how these differ in bilateral HA users from age-matched NH listeners. Further, measurements of binaural pitch fusion and absolute localization acuity were conducted on the same subject groups that participated in the speech-on-speech masking experiment. We explored whether variability in pitch fusion and localization acuity could explain the variability in VGRM and SRM, respectively. In order to check that these correlations are truly due to binaural processing, speech-on-speech masking experiments were repeated in two monaural (left ear and right ear) listening conditions, and their results were compared with those in the bilateral listening conditions. Our primary hypothesis was that broad binaural pitch fusion would be associated with reduced benefit from the voice-gender difference cue, and conversely that narrow binaural pitch fusion would be associated with a greater advantage in the use of this cue. In other words, the benefit from the voice gender difference cue (VGRM) would be negatively correlated with the binaural pitch fusion ranges. We also hypothesized a negative correlation between sound localization acuity and masking release by spatial separation (SRM). That is, poor localization acuity would be associated with reduced SRM, and conversely that acute localization acuity would be associated with a greater advantage in SRM. Finally, we expected that no correlations would be observed with the monaural listening conditions.

## Materials and methods

### Participants

All measurements were conducted according to the guidelines for the protection of human subjects as set forth by the Institutional Review Boards (IRBs) of both Oregon Health and Sciences University and the Portland VA Medical Center, and the methods employed were approved by these IRBs. Twenty-three adult subjects, consisting of eleven NH listeners ranging in age from 36 to 67 years (mean and standard deviation (std) = 50.0 ± 9.9 years; 7 females), twelve bilateral HA users ranging in age from 30 to 75 years (mean and std = 53.8 ± 16.7 years; 10 females; Table 1), participated in this study. A Kruskal–Wallis *H*-test showed that there were no significant age differences between these two listener groups [H(1) = 1.817, *p* = 0.611]. All subjects were native English speakers and screened for normal cognitive function using the 10-min Mini Mental Status Examination (MMSE) with a minimum score of 27 out of 30 required to qualify (Folstein et al., 1975; Souza et al., 2007), ruling out cognitive impairment that would potentially influence performance.

**TABLE 1:** Demographic information for hearing-aid (HA) users: age, sex, etiology of hearing loss, and reference ear.

Subject ID	Age (years)	Sex	Etiology of hearing loss	Reference ear
H1	75	Male	Unknown	Right
H2	30	Female	Genetic	Right
H3	39	Female	Genetic	Right
H4	67	Female	Genetic	Right
H5	34	Female	Unknown	Right
H6	39	Male	Genetic	Right
H7	71	Female	Unknown	Left
H8	47	Female	Noise	Left
H9	67	Female	Unknown	Right
H10	73	Female	Unknown	Left
H11	43	Female	Genetic	Left
H12	60	Female	Unknown	Left
M	53.8			
SD	16.7			

**FIGURE 1:**
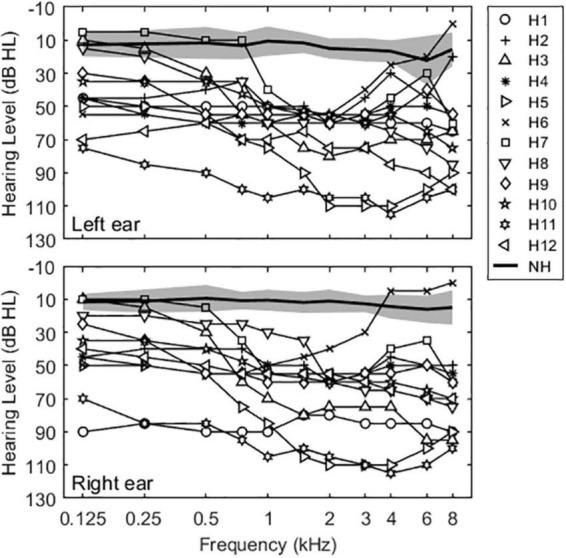
Unaided audiograms for the NH and bilateral HA subjects in this study. Solid thin lines show individual thresholds for bilateral HA users. Solid thick lines and shaded areas represent averaged thresholds and standard deviations for NH subjects.

Normal hearing was defined as air conduction thresholds ≤25 dB hearing level (HL) from 125 to 4000 Hz. Mean pure-tone averages at octave interval frequencies between 125 and 4000 Hz for NH subjects were 12.6 ± 2.2 dB HL for the left ear and 11.5 ± 1.4 dB HL for the right ear. Bilateral HA users had moderate to severe hearing losses in both ears and relatively symmetric losses between ears, with the exception of subject H1. Mean pure-tone averages were 56.5 ± 10.8 and 57.7 ± 10.5 dB HL for left and right ears, respectively. Figure 1 shows group-averaged audiograms for NH subjects (thick solid lines) and individual audiograms for bilateral HA subjects (lines with open symbols) for left and right ears.

All bilateral HA users were required to have at least 1 year of experience with bilateral HA use and have monaural word intelligibility scores of 65% or higher on the Consonant Nucleus Consonant (CNC) word test with both devices. For the speech-on-speech masking experiment and the sound localization acuity experiment, all HA users used lab loaner HA devices (Phonak Ambra). All extra processing features for hearing devices were disabled, including adaptive/automatic gain control, frequency lowering, directional microphones, and noise reduction. HAs were verified to meet NAL-NL2 (National Acoustics Laboratories-Non-Linear2, Australia) targets (speech stimuli at 50, 65, and 75 dB SPL) using real-ear measurements in order to provide suitable amplification for a subject’s hearing loss, and all subjects met the target criteria. In both subject groups, tympanometry was also conducted to verify normal middle ear function. Additional details of etiology of hearing loss of the HA users are shown in Table 1. All subjects were paid an hourly wage and completed all experiments in between four to seven sessions of 2–3 h each. No prior experience with psychophysical research was required for participation; however, practice tutorials (20–30 min) were provided to all subjects in order to assure familiarity with the procedures.

### Stimuli and procedures

Three main experiments were conducted in this study: speech recognition threshold measurement in competing speech, binaural pitch fusion range measurement, and localization acuity measurement. The measurements of both speech recognition threshold and localization acuity were conducted in the anechoic chamber located at the National Center for Rehabilitative Auditory Research (NCRAR). The measurement of binaural pitch fusion range was conducted in a double–walled, sound attenuated booth at the Oregon Hearing Research Center (OHRC). All statistical analyses were conducted in SPSS (version 25, IBM).

#### Speech-on-speech masking measurement

All speech stimuli were digitally processed in MATLAB to have a sampling rate of 44.1 kHz. Stimuli were presented through a bank of three eight-channel amplifiers (Ashlys/ne4250) and 24 frequency-equalized loudspeakers calibrated by a Brüel and Kjaer sound level meter. The loudspeakers were arranged in a circle in the horizontal plane with 15° increments surrounding the listener and equidistant at 2 m from the listener’s head.

All speech stimuli were drawn from the Coordinate Response Measure (CRM; Bolia et al., 2000) speech corpus, which consists of sentences in the form “*Ready* [*call sign*] *go to* [*color*] [*number*] *now*.” In this study, speech stimuli were presented with a 20% slower speaking rate than the original CRM corpus stimuli because some HA users had difficulties in understanding target-only stimuli at the original speaking rate. A custom MATLAB implementation of a modified pitch synchronous overlap add (PSOLA) technique (Moulines and Laroche, 1995) was used to time-stretch CRM sentences by 20%. There are eight possible *call signs* (Arrow, Baron, Charlie, Eagle, Hopper, Laker, Ringo, and Tiger), and 12 keywords: four *colors* (red, green, white, and blue) and the *numbers* (1–8). All possible combinations of the *call signs*, *colors*, and *numbers* were spoken by four male (*F*_0_ = 100 ± 7 Hz) and four female talkers (*F*_0_ = 204 ± 12 Hz). Note that fundamental frequency (*F*_0_), which represents the voice pitch, was estimated using the cepstrum algorithm in MATLAB where the output is the Fourier transform of the log of the magnitude spectrum of the input waveform (Flanagan, 1965). *F*_0_ for each talker was averaged across all of that talker’s CRM speech stimuli.

Each subject was presented with three simultaneous sentences from the CRM corpus (1 target and 2 simultaneous maskers). Subjects identified keywords associated with one target sentence while attempting to ignore two masker sentences. Target speech stimuli were presented from directly in front of the listener with a fixed sound presentation level of 60 dB SPL. Masker speech stimuli were presented in one of two spatial configurations: co-located (target at 0°, maskers at 0°) or 60° symmetrical separations (target at 0°, maskers at ± 60°). Only symmetrical target-masker separation conditions were considered in order to minimize availability of the better ear cue (monaural head shadow effect; Shaw, 1974; Kidd et al., 1998) and maximize reliance on spatial cues or voice-gender cues for source segregation.

These two spatial conditions were tested with four different gender combinations of target and maskers: MM (male target, male maskers), MF (male target, female maskers), FF (female target, female maskers), and FM (female target, male maskers), for a total of 2 × 4 = 8 conditions. In each trial, the subject was instructed to face the front speaker and attend to the target sentence, always identified here by the *call sign* “Charlie,” and indicate the target *color* and *number* keywords from the 32 possible *color*/*number* combinations. The masker sentences had exactly the same form as the target but a different *call sign*, *color*, and *number*, randomly selected on each trial. The one target and two masker sentences were randomized from eight talkers (four males and four females) for each target-masker gender combination at each trial, and they were temporally aligned at the beginning and were roughly the same total duration.

Responses were obtained using a touch screen monitor located on a stand within arm’s reach of the listener seated in the middle of the anechoic chamber. The monitor was directly in front of the listener but below the plane of the loudspeakers. Subjects were asked to look straight ahead and to hold their heads stead during a stimulus presentation. Feedback was given after each presentation in the form of “Correct” or “Incorrect.” Approximately one second of silence followed the response being registered, prior to the next stimulus presentation.

The masker sound presentation level was adaptively varied at each trial to find the target-to-masker ratio (TMR), or the masker level yielding 50% correct recognition of both target *color* and *number* (i.e., 1/32 chance), using a one-up/one-down procedure (Levitt, 1971). The initial level for the masker sentence was set at 30 dB SPL and increased in level by 5 dB for each correct response until an incorrect response occurred, then decreased in level for each incorrect response until a correct response, and so on. This was repeated until three reversals in direction were obtained, at which point the step size was changed to 1 dB and six more reversals were measured. The TMR was estimated as the average of the last six reversals. Note that TMR indicates the difference in level between the target and each masker in the symmetrical target-masker separation conditions, while signal-to-noise ratio (SNR) refers to difference between the target and the combined masker level. For example, if the target level is 60 dB SPL and each masker is also 60 dB SPL, the TMR would be 0 dB, and the overall SNR would be approximately −3 dB. All subjects were tested in binaural listening conditions and in both monaural listening conditions with the non-test ear plugged and muffed. Thresholds were averaged over three separate runs for each condition.

#### Binaural pitch fusion measurement

All stimuli were digitally generated at a sampling rate of 44.1 kHz with MATLAB, delivered using an ESI Juli sound card, TDT PA5 digital attenuator and HB7 headphone buffer, and presented over Sennheiser HD-25 headphones. Headphone frequency responses were equalized using calibration measurements obtained with a Brüel and Kjaer sound level meter with a 1-inch microphone in an artificial ear.

Prior to the binaural fusion range measurements, loudness balancing was conducted sequentially across frequencies and across ears using a method of adjustment. For both listener groups, 300-ms tones at 0.125, 0.25, 0.375, 0.5, 0.625, 0.75, 0.875, 1, 1.25, 1.5, 2, 3, and 4 kHz in the reference ear were initialized to “medium loud and comfortable” levels corresponding to a 6 or “most comfortable” on a visual loudness scale from 0 (no sound) to 10 (too loud). Loudness for the comparison ear was then adjusted for each frequency to be equally loud to a tone in the reference ear during sequential presentation across the ears, based on subject feedback. Here, all loudness balancing adjustments were repeated with a fine attenuation resolution (0.1 dB steps for bilateral HA and 0.5 dB steps for NH listeners) until equal loudness was achieved with all comparison sequences within and across ears, with a reference to a 500-Hz tone in the reference ear. The averaged comfortable sound levels were 65 ± 4/65 ± 4.1 dB sound pressure level, SPL (left/right ear) for NH listeners and 90 ± 1.4/91 ± 1.7 dB SPL (left/right ear) for bilateral HA users. The frequencies and order of presentation were randomized to minimize the effect of biases such as time-order error and underestimation or overestimation of the loudness (Florentine et al., 2011). This loudness balancing procedure was performed to minimize use of level-difference cues and maximize focus on pitch differences as the decision criteria. Using the same program, each ear was then checked for poor within-ear pitch ranking ability by asking subjects to rank which tone was higher in pitch for all frequency combinations.

Binaural pitch fusion range measurements were then performed to measure the fusion ranges over which dichotic pitches were fused with dichotic 1500-ms harmonic tone complexes. The method of constant stimuli procedure was used: the reference stimulus was fixed in the designated “reference ear,” and the contralateral, comparison stimulus was varied across trials. For NH listeners, the reference ear was randomized. For bilateral HA users, if one ear had poor within-ear frequency discrimination as assessed during the loudness balancing procedure, that ear was assigned to be the reference ear so that the resolution of comparison stimulus testing would be maximized using the contralateral better ear, instead of limited by the worse ear. The reference fundamental frequency (*F*_0ref_) was fixed at 200 Hz, and the comparison stimuli consisted of other harmonic complexes with fundamental frequencies (*F*_0comp_) sampled around the reference with 1/64 to 1/16 octave steps and varied pseudo-randomly across trials. The number of harmonic components was fixed at four.

At each trial, subjects were asked to indicate whether they heard a single fused sound or two different sounds through a touch screen monitor. If a single sound was heard, subjects were instructed to indicate whether they heard that sound as a single fused sound (“Same”). If two different sounds were heard, subjects were instructed to indicate which ear had the higher pitch (“Left higher” or “Right higher”) as a check of whether two sounds were really heard. A “Repeat” button was also provided to allow subjects to listen to the stimuli again. No feedback was given during the run. Binaural pitch fusion ranges were averaged over three separate runs.

#### Localization acuity measurement

Three Gaussian noise-band stimuli with 500-ms duration were generated with sixth-order Butterworth filter and processed in MATLAB to have a sampling rate of 44.1 kHz. The broadband stimulus was band-pass noise filtered between 125 and 8000 Hz, and two narrowband stimuli were band-pass noises centered at 500 and 3000 Hz with 1/3-octave-wide bands. All stimuli were presented through the same 24-loudspeaker array system and equipment configuration as used in the speech-on-speech masking experiment.

Prior to the localization acuity measurements, threshold estimates of “quiet detection threshold” were performed to ensure the audibility of each noise stimulus. A one-up/two-down adaptive procedure tracking the 70.7% correct point (Levitt, 1971) was used with a four-interval (two-cue, two-alternative). On each trial, the target sound was assigned to the second or third interval with equal probability, and no signal was presented in the first and the fourth intervals. The initial level was set at 50 dB SPL and decreased in level for two consecutive correct responses until an incorrect response occurred, then increased in level for each incorrect response until a correct response, and so on. This was repeated until three reversals in direction were obtained, at which point the step size was decreased by half for each reversal. The average of the last six reversals with a 1-dB step size was used to estimate thresholds. The averaged quiet threshold levels were 21 ± 3.2/24 ± 5.1/25 ± 5.3 dB SPL (broadband/500-Hz band-pass noise/3000-Hz band-pass noise) for NH listeners and 32 ± 9.4/40 ± 8.7/43 ± 7.2 dB SPL (broadband/500-Hz band-pass noise/3000-Hz band-pass noise) for bilateral HA users.

Localization acuity measurements were then performed with the method of constant stimuli procedure for each stimulus condition: three presentations of the 24 speakers in random order (i.e., 72 trials for each stimulus condition). The stimulus level was fixed at 30 dB sensational level (SL). Subjects were asked to look straight ahead and to hold their heads steady during a stimulus presentation and asked to identify the location of the sound through the touchscreen (a circle with a radius of 5 cm without a visual representation of all speakers) after stimulus presentation. No feedback was given during the run. Localization acuity was averaged over three separate runs for each stimulus condition.

## Results

### Effects of voice-gender differences and spatial separation on speech recognition thresholds in noise

**FIGURE 2:**
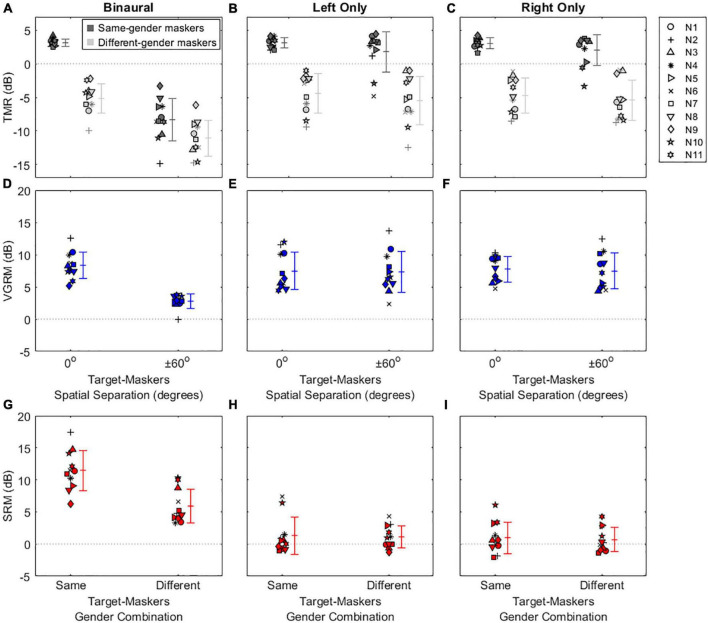
Individual and average target-to-masker ratio (TMR) thresholds and voice gender release from masking (VGRM) and spatial release from masking (SRM) for NH listeners. The left, middle, and right columns refer to the binaural, left only, and right only listening conditions, respectively. The upper panels show the TMR thresholds **(A–C)** as a function of target-masker spatial separation (0 and ± 60°). Dark-shaded and light-shaded symbols indicate TMR thresholds for the same-gender masker and the different-gender masker conditions, respectively. The middle panels show VGRMs **(D–F)** as a function of target-masker spatial separation (0 and ± 60°). The lower panels show SRMs **(G–I)** as a function of target-masker gender combination (same-gender and different-gender). Error bars represent standard deviation around the mean. Horizontal dotted lines represent reference zero values.

**FIGURE 3:**
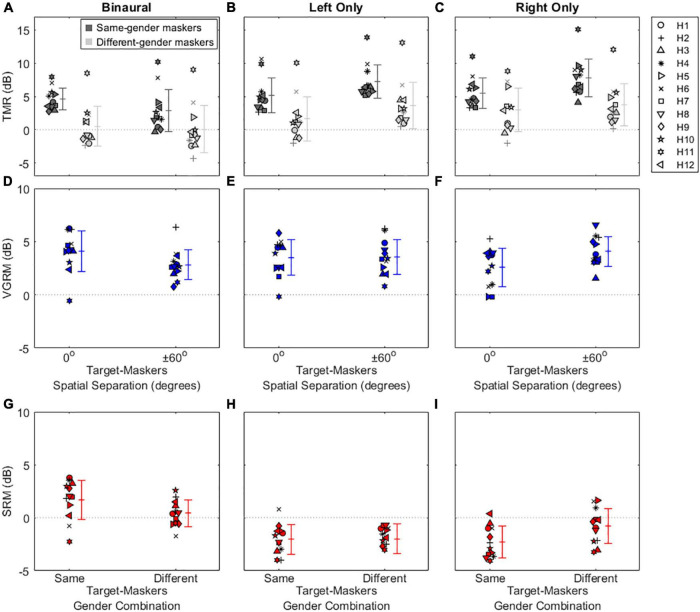
Individual and average target-to-masker ratio (TMR) thresholds and voice gender release from masking (VGRM) and spatial release from masking (SRM) for bilateral HA users. Plotted as in Figure 2, with different scales on the *y*-axis.

Figures 2, 3 show the results of the speech-on-speech masking experiment for NH and HA user groups, respectively. Note that the TMR thresholds of the two same-gender conditions (MM and FF) were similar at each spatial configuration in both groups, as were those of the two different-gender conditions (MF and FM), and these TMR threshold similarities between talker-masker gender combinations were also reported in the previous studies (Gallun et al., 2013; Oh et al., 2021) that used the same experimental setup as the current study. Thus, the TMR thresholds averaged in the same-gender vs. the different-gender conditions were used for all plots and statistical analyses in this study.

The top row of Figure 2 shows individual and mean TMR thresholds as a function of target-maskers spatial separation (0 and ± 60°) for three listening conditions (binaural, left only, and right only) in NH listeners. Generally, smaller or more negative TMR thresholds indicate better (or improved) speech recognition ability in noise. In the binaural listening condition, the results show that the same-gender condition (3.16 ± 0.56 dB) exhibits larger (poorer) TMR thresholds than the different-gender condition (−5.18 ± 2.19 dB) in the co-located target-maskers spatial configuration. A similar trend was observed in the spatially (± 60°) separated configuration (the same-gender condition: −8.31 ± 3.14 dB; the different-gender condition: −11.09 ± 2.65 dB). In both spatial configurations, the lower TMR values for the different-gender conditions relative to the same-gender conditions are indicative of the amount of VGRM, which shows how much speech recognition thresholds in noise are improved by differences in gender between the target and maskers. The amount of VGRM (Figure 2D) was calculated by the difference in TMR thresholds between same-gender (dark-gray symbols) and different-gender (light-gray symbols) conditions at each spatial configuration. The VGRM for NH listeners (Figure 2D) ranged between −0.11 and 12.52 dB, and the mean VGRM was greater in the co-located spatial configuration (8.34 ± 2.07 dB) than in the spatially separated configuration (2.79 ± 1.10 dB).

Another interesting finding in NH listeners is that spatial separation of the maskers to ± 60° relative to the target at 0° led to smaller (better) TMR thresholds for all target-masker gender combinations. This reduction is indicative of the amount of SRM, which shows how much speech recognition thresholds are improved by spatial separation of the talker from the maskers. The amount of SRM (Figure 2G) is defined as the spatial separation benefits at each target-masker gender combination [i.e., differences between dark-gray (or light gray) symbols at 0° and at ± 60° in Figure 2A]. The SRM for NH listeners ranged between 3.42 and 17.29 dB, and the mean SRM was greater in the same-gender target-maskers combination (11.47 ± 3.12 dB) than in the different-gender combination (5.91 ± 2.61 dB).

Compared to the binaural listening condition, the two monaural listening conditions elicited TMR threshold changes, especially in the spatially separated target-maskers configuration, and thus different results in VGRM and SRM. First, the TMR thresholds in the left-only (Figure 2B) and right-only (Figure 2C) listening conditions were similar to those in the binaural listening condition (Figure 2A) at the co-located target-maskers configuration (left only: 3.11 ± 0.78 dB same-gender/−4.38 ± 2.92 dB different-gender; right only: 3.02 ± 0.82 dB same-gender/−4.72 ± 2.64 dB different-gender). However, the monaural TMR thresholds were essentially unchanged compared to the co-located condition when the target and maskers were spatially separated (left only: 1.81 ± 2.99 dB same-gender/−5.48 ± 3.60 dB different-gender; right only: 2.06 ± 2.32 dB same-gender/−5.40 ± 2.99 dB different-gender). The masking release results in the two monaural listening conditions show that the VGRM remained steady at around 8 dB regardless of spatial separation between target and maskers (Figures 2E,F), while SRM was decreased to near zero regardless of target-maskers gender differences (Figures 2H,I).

The results in the top row of Figure 3 show that bilateral HA users exhibited overall poorer speech recognition performance (i.e., more positive TMR thresholds with a range between −4.38 and 15.09 dB) throughout all listening conditions compared to NH listeners (TMR thresholds with a range between −14.85 and 4.43 dB). Interestingly, spatial separation between target and maskers didn’t improve TMR thresholds for HA users even in the binaural listening condition (differences between 0 and ± 60° in the same-colored symbols). The mean SRMs for bilateral HA users (Figure 3G) were 1.70 ± 1.84 dB and 0.41 ± 1.24 dB for the same-gender and different-gender talker combinations, respectively. In contrast, benefits from voice-gender differences existed in both spatial separation configurations, thus positive mean VGRMs (Figure 3D) were observed (4.11 ± 1.89 dB for the 0° and 2.83 ± 1.42 dB for ± 60° spatial separations). In the two monaural listening conditions, the SRM performance was more degraded (−2 dB shown in Figures 3H,I) than in the binaural listening conditions; however, the VGRM performance was remained steady at around 4 dB (Figures 3E,F).

Since the primary goal of this study was to investigate masking release by voice-gender differences (VGRM) and spatial separations (SRM), only the masking release data were analyzed in each masking release type using linear mixed model (LMM) analyses with the amount of masking release (VGRM or SRM) as a dependent variable, the subject group (NH vs. bilateral HA), the listening conditions (binaural vs. left only vs. right only), and the target-maskers conditions (spatial separation for VGRM: 0° vs. ± 60°; gender difference for SRM: same-gender vs. different gender) as fixed effects, and the subject as a random effect. The results for both VGRM and SRM showed significant main effects of all fixed factors (*p* < 0.006 for all cases) and significant interactions between any two combinations of the fixed factors (*p* < 0.006 for all cases). *Post-hoc* pairwise comparisons using Bonferroni correction were computed to better understand the interaction between those fixed factors. The results demonstrated that the VGRM at the ± 60° in the binaural listening condition was significantly lower than all other VGRMs in the NH listeners (*p* < 0.001 for all cases), but no VGRMs were significantly different in bilateral HA users (*p* = 1.000 for all cases). In addition, the results demonstrated that the SRM in NH listeners was significantly higher in the binaural listening condition than in two monaural listening conditions (*p* < 0.001 for all cases), and the same-gender target-maskers combination elicited a significantly higher masking release than the different-gender combination in the binaural listening condition (*p* < 0.001). A similar binaural listening benefit in SRM was also observed in the bilateral HA user group (*p* < 0.05 for all cases), but the SRMs were not significantly different between the two target-maskers gender combinations in the binaural listening condition (*p* = 1.000). Please see the Supplementary material for the detailed LMM specifications and results.

### Binaural pitch fusion and its relationship with voice gender release from masking

**FIGURE 4:**
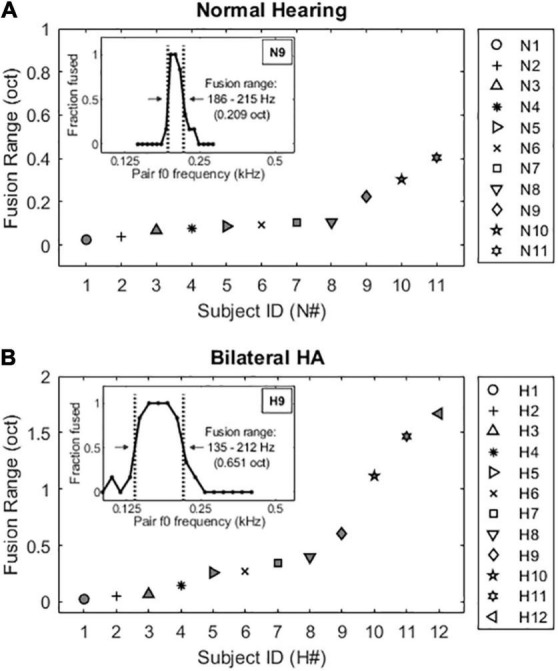
Individual harmonic tone fusion range results in an octave scale for NH listeners **(A)** and bilateral HA users **(B)**. A sample fusion function inset within each panel illustrates the fusion ranges of the 50% points (vertical dotted lines) on the fusion function.

Figure 4 shows individual harmonic tone fusion range results for NH listeners (Figure 4A) and bilateral HA users (Figure 4B). As shown in the example fusion functions in the insets of Figure 4, fusion functions were computed as the averages of the subject responses to the multiple (six to seven) presentations of each reference and comparison stimulus pair, expressed as a function of comparison tone fundamental frequency. Values near 0 indicate comparison stimuli that did not often fuse with the reference stimulus (were heard as two sounds), while values near 1 indicate comparison stimuli that were often fused with the reference stimulus (were heard as one sound). Vertical dotted lines indicate 50% points on the fusion function, and the fusion range was defined as the range between these two lines (horizontal arrows), i.e., frequencies were fused more than 50% of the time. Fusion range is thus a measure of the breadth of fusion. The NH subjects (Figure 4A) exhibited narrow harmonic tone fusion ranges (0.14 ± 0.12 octaves), while bilateral HA users (Figure 4B) showed significantly broader harmonic tone fusion ranges [0.53 ± 0.57 octaves; t(21) = −2.25, *p* = 0.036].

**FIGURE 5:**
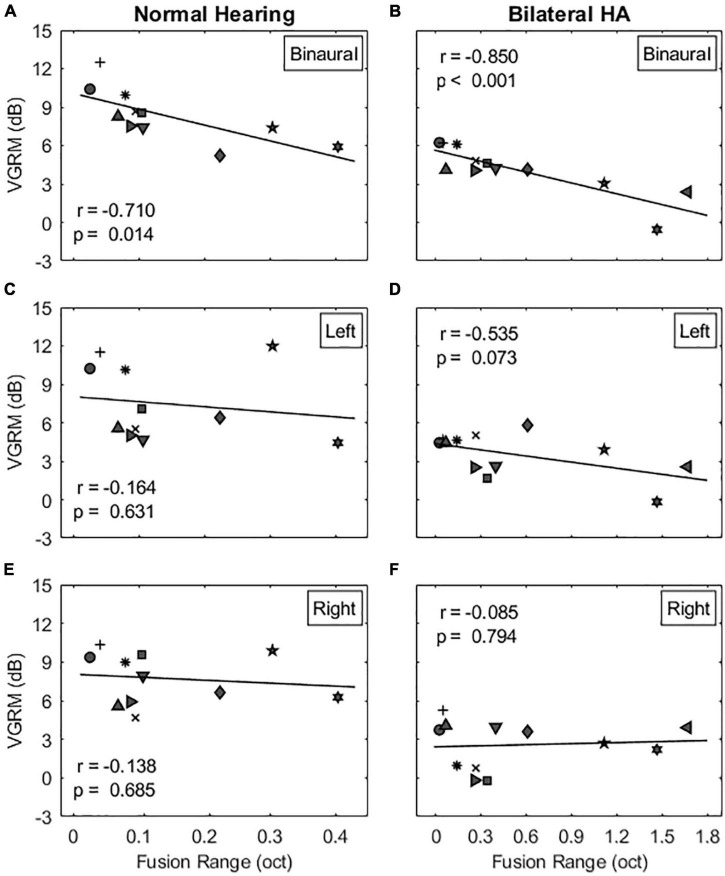
Correlations between voice gender release from masking (VGRM) and binaural pitch fusion range for the co-located target-maskers configuration. The left and right columns show the correlation results for NH and bilateral HA user groups, respectively. The panels **(A–F)** show the correlation results for the binaural, left, and right listening conditions, respectively. Table 2 shows the correlation results for the spatially separated target-maskers configuration.

The next step was to determine whether VGRM, the release from masking due to voice-gender differences between target and maskers, is related to the width of binaural pitch fusion. Multiple regression analyses were conducted to measure a linear relationship between two variables. Figure 5 shows individual VGRMs plotted as a function of fusion ranges in the co-located target-maskers configuration for NH listeners (left column) and bilateral HA users (right column). In the binaural listening condition, VGRM was significantly correlated with the fusion range in both subject groups (NH listeners: *r* = −0.710, *p* = 0.014 in Figure 5A; bilateral HA users: *r* = −0.850, *p* < 0.001 in Figure 5B). In other words, listeners with narrow binaural pitch fusion ranges had larger VGRM (larger differences in TMR thresholds between same-gender and different-gender maskers) than did listeners with broad fusion. However, this negative correlation between VGRM and fusion range was eliminated in the two monaural listening conditions in both listener groups (see Figures 5C–F: *p* > 0.073 for all cases). Note also that some listeners with broad fusion had greater VGRM in one or both monaural conditions compared to the binaural condition (e.g., N10 and H9, indicated by star and diamond symbols in Figures 5A–E, respectively). As provided in Table 2, no significant correlation was observed in the spatially separated target-maskers configuration as well (*p* > 0.163 for all cases).

**TABLE 2:** Regression coefficients between voice gender release from masking (VGRM) and binaural pitch fusion range widths for NH and bilateral HA user groups in each spatial separation and listening condition.

Target-maskers spatial separation	Listening condition	Correlation *r*-values (significance)	
		NH	Bilateral HA
0 degree	Binaural	−0.710 (0.014)*	−0.850 (<0.001)***
	Left only	−0.164 (0.631)	−0.535 (0.073)
	Right only	−0.138 (0.685)	−0.085 (0.794)
± 60 degree	Binaural	−0.428 (0.195)	−0.586 (0.063)
	Left only	−0.338 (0.310)	−0.260 (0.414)
	Right only	−0.350 (0.291)	−0.249 (0.435)

Correlation values in bold face indicate significant results (***p < 0.001; *p < 0.05).

### Localization acuity and its relationship with spatial release from masking

**FIGURE 6:**
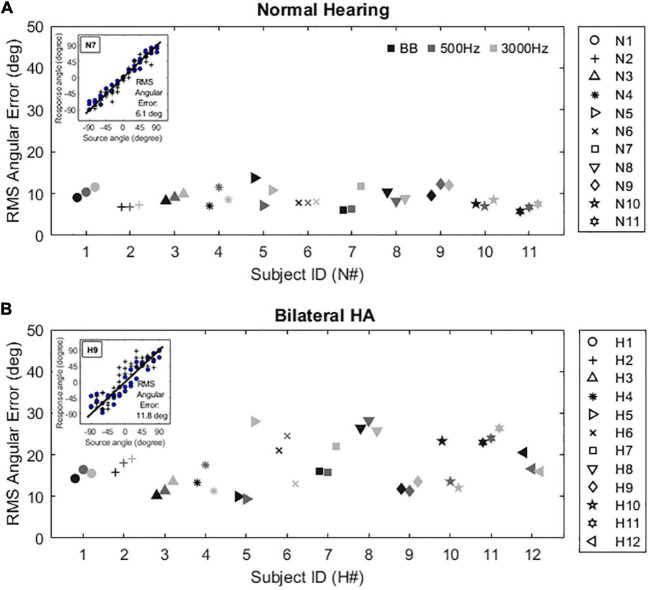
Individual localization acuity results with three different stimuli (BB: broadband, 500 Hz: band-passed noises centered at 500 Hz, 3000 Hz: band-passed noises centered at 3000 Hz) for NH listeners **(A)** and bilateral HA users **(B)**. A sample subject response inset within each panel illustrates the mean root mean square (RMS) angular error calculated by the difference between the perfect localization (diagonal line) and the listener’s response (symbols) angles. The circle and plus symbols indicate the subject’s responses to any given source locations in the front and rear source fields, respectively.

Figure 6 shows individual minimum audible angle results for NH listeners (Figure 6A) and bilateral HA users (Figure 6B). Example localization scatter plots were shown in the insets of Figure 6. The subject’s response angles were plotted as a function of the source angles, and ideal performance would be represented by all points lying on the diagonal lines. The root-mean-square (RMS) angular errors were calculated to quantify a subject’s accuracy in localizing sound sources (Lorenzi et al., 1999). It should be noted that the circle and plus symbols in the insets of Figure 6 indicate the subject’s responses to any given source locations in the front and rear source fields, respectively, and that front-back confusions were excluded for estimating the absolute localization ability in this study. The NH subjects (Figure 6A) exhibited fine localization acuity with all stimuli tested in this study (broadband: 5.75 to 13.75°; 500-Hz band-pass noise: 6.2 to 12.35°; 3000-Hz band-pass noise: 7.25 to 11.65), while bilateral HA users (Figure 6B) showed significantly poorer localization acuity [broadband: 10 to 26.4°; 500-Hz band-pass noise: 9.4 to 28.2°; 3000-Hz band-pass noise: 11.2 to 26.35 degree; t(48.5) <−4.61, *p* < 0.001 for all stimulus cases]. The localization acuity was not significantly different across the stimulus types for each subject group [NH: t(42) >−0.760, *p* = 1; bilateral HA: t(42) >−0.619, *p* = 1].

**FIGURE 7:**
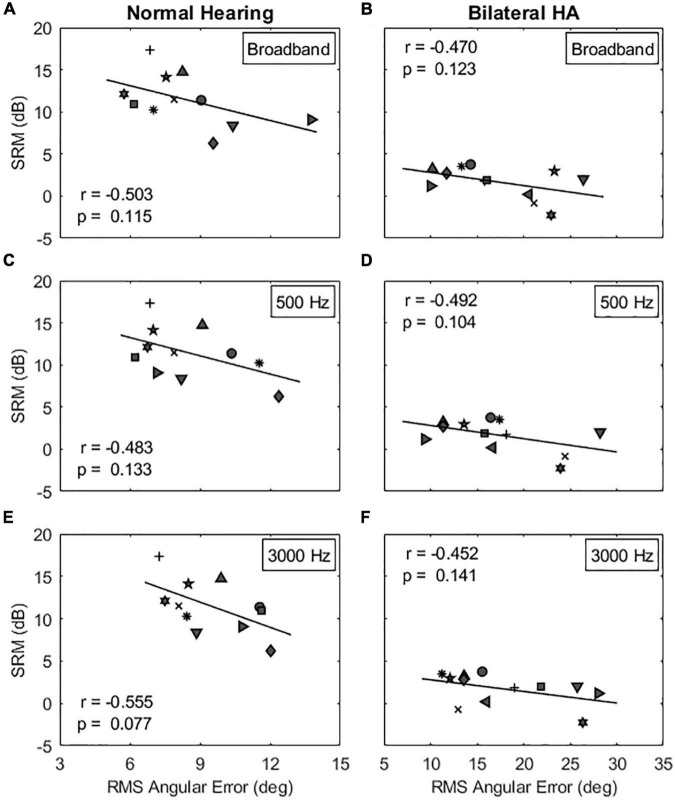
Correlations between spatial release from masking (SRM) and localization acuity for the same-gender target-maskers condition. The left and right columns show the correlation results for NH and bilateral HA user groups, respectively. The panels **(A–F)** show the correlation results for the broadband, 500 and 3000 Hz stimulus conditions, respectively. Table 3 shows the correlation results for the different-gender target-maskers condition.

The next step was to determine whether SRM, the release from masking due to spatial separation between target and maskers, is related to the absolute localization ability quantified as the RMS angular error. Multiple regression analyses were conducted to measure a linear relationship between two variables. Figure 7 shows individual SRMs plotted as a function of RMS angular errors in the same-gender target-maskers combination for NH listeners (left column) and bilateral HA users (right column). Results show that the SRM was correlated, but the correlation was not statistically significant, with the RMS angular errors for all stimuli tested in this study (*p* > 0.077). In other words, there was a tendency for listeners with sharp localization acuity to have larger SRM (larger differences in TMR thresholds between co-located and spatially separated maskers) compared to listeners with poor localization acuity. In addition, this correlation was reduced in the different-gender target-maskers combination (not shown). The model summary of the regression analysis is provided in Table 3.

**TABLE 3:** Regression coefficients between spatial release from masking (SRM) and localization acuity for NH and bilateral HA user groups in each stimulus and gender-combination conditions.

Target-maskers gender combination	Stimulus type	Correlation *r*-values (significance)	
		NH	Bilateral HA
Same-gender	Broadband	−0.503 (0.115)	−0.470 (0.123)
	500 Hz	−0.483 (0.133)	−0.492 (0.104)
	3000 Hz	−0.555 (0.077)	−0.452 (0.141)
Different-gender	Broadband	−0.210 (0.491)	−0.128 (0.692)
	500 Hz	−0.379 (0.536)	−0.228 (0.477)
	3000 Hz	−0.236 (0.456)	−0.263 (0.408)

## Discussion

The ability to segregate a target talker from competing masker talkers is important for speech perception in multi-talker listening environments. The current study measured speech-on-speech masking performance by varying voice-gender differences and spatial separation cues between target and maskers in both NH listeners and bilateral HA users, and examined how this performance relates to binaural pitch fusion range and localization acuity.

The results from NH listeners showed that VGRM, the average masking release *via* voice-gender differences, was maximized at 8.34 dB in the co-located spatial configuration and reduced to 2.79 dB in the separated spatial configuration. Similarly, SRM, the average masking release *via* talker spatial separation, was maximized at 11.47 dB when the target was presented with the same-gender maskers and reduced to 5.91 dB when the different-gender target-maskers were presented. Consistent with previous studies, these findings demonstrate a trading relationship between the perceptual weights applied to voice-gender difference and those to spatial separation cues. This trading relationship of masking release was also partially discussed in previous literature (Misurelli and Litovsky, 2012, 2015; Gallun and Diedesch, 2013; Gallun et al., 2013; Oh et al., 2021). The current study results also indicate that this trading relationship is eliminated in monaural listening conditions. SRM was minimized at around 1 dB regardless of the talkers’ gender difference cue, while VGRM was maintained at around 8 dB regardless of the talkers’ spatial separation cue. Hence, the trading relationship between SRM and VGRM appears to be related to the presence of binaural cues.

The results from bilateral HA users showed that average VGRM was 4.11 and 2.83 dB for co-located and spatially separated conditions, while average SRM was 1.7 and 0.41 dB for the same-gender and different-gender maskers. As in NH listeners, a trading relationship was observed between the two masking release types, though not as pronounced. In addition, both voice gender difference and spatial separation benefits were reduced in HA users compared to NH listeners.

Previous studies have reported that reduced masking release performance observed in bilateral HA users could be attributed to reduced ability to access monaural spectro-temporal cues and/or binaural cues caused by either aging or hearing loss (Best et al., 2011, 2012; Gallun et al., 2013; Füllgrabe et al., 2015; Srinivasan et al., 2021). In this study, we also conducted multiple regression analyses to find a linear relationship between two different types of masking releases (VGRM and SRM; combined both NH and HA subjects’ data) and subject factors (e.g., age and degree of hearing loss). The results showed that the pure-tone average (PTA from 125 and 4000 Hz) accounted for more than 18% (*R*^2^ predictor, *p* < 0.045) of the variance in both VGRM and SRM; however, age couldn’t explain VGRM and SRM variances (*p* > 0.227). The model summary of the regression analysis is provided in Table 4. However, as will be discussed, broad binaural pitch fusion and poor sound localization abilities might be other factors reducing overall SRM and VGRM.

**TABLE 4:** Regression coefficients for masking release by voice-gender differences (VGRM) and spatial separation (SRM), binaural pitch fusion range, and absolute localization acuity predicted by age and pure tone average (PTA).

Measurement	Condition	Predictor variable	Correlation *r*-values (significance)
VGRM	Co-located	Age	−0.262 (0.227)
	target-maskers	PTA	−0.713 (<0.001)***
	Spatially separated	Age	−0.254 (0.243)
	target-maskers	PTA	−0.537 (0.008)**
SRM	Same-gender	Age	−0.126 (0.565)
	target-maskers	PTA	−0.636 (0.001)**
	Different-gender	Age	−0.092 (0.677)
	target-maskers	PTA	−0.423 (0.045)*
Binaural pitch	–	Age	0.200 (0.371)
fusion range		PTA	0.534 (0.009)**
Absolute	Broadband	Age	0.227 (0.297)
localization		PTA	0.627 (0.001)**
acuity	500 Hz	Age	0.087 (0.692)
		PTA	0.588 (0.003)**
	3000 Hz	Age	0.088 (0.690)
		PTA	0.763 (<0.001)***

Correlation values in bold face indicate significant results (***p < 0.001; **p < 0.01; *p < 0.05).

One likely reason for the reduced SRM, though, for bilateral HA users is that they have limited access to binaural cues on the horizontal plane such as ITD and ILD cues. Previous studies have shown that ITD sensitivity is particularly important for localization performance and speech perception in noise (Gallun et al., 2005; Gallun and Diedesch, 2013; Gifford et al., 2013, 2014; Swaminathan et al., 2016; Ellinger et al., 2017). Phase-locking and ITD sensitivity can both be impaired with hearing loss (Henry and Heinz, 2013; Dai et al., 2018). In addition, bilateral HA users have reduced access to ongoing ITD cues, because the hearing devices are not designed to coordinate their timing of stimulation of the auditory nerves across the ears (Brown et al., 2016). Thus, they do not communicate their processing schemes (such as compression ratio) across the devices, especially for old hearing devices, which could alter ILDs (Byrne and Noble, 1998; Wiggins and Seeber, 2013). To minimize any potential interaural cue distortion, the current study used symmetrical target-masker configurations (co-location and ± 60° separation) so that the image of both target and masker signals can appear in front, as opposed to the left or right due to reduced ILD, and all additional processing features for hearing devices were disabled to avoid altered ILD cues. Note that in this study, effects of head shadow were also minimized due to the symmetrical target-masker configuration. In addition, all HA users used lab loaner HA devices (Phonak Ambra) with all extra processing features disabled. Due to lack of acclimation, overall performance may be reduced with the loaner devices compared with the subjects’ own hearing devices. However, for evaluation of VGRM and SRM in this study, it is important to disable these extra processing features, which often include noise reduction and directional microphones.

There was also significant variation in listeners’ masking release performance for both NH and HI listeners. The findings of this study show that, as hypothesized, binaural pitch fusion range is a strong predictor for variation in VGRM. In contrast, localization ability does not seem to predict variation in SRM, though a non-significant trend was observed.

Regarding the relationship of binaural fusion to VGRM, a strong negative correlation was observed. Previous studies have found that differences in age or hearing loss (alone or in combination) can explain some of the variance across subjects (Glyde et al., 2013; Besser et al., 2015). The proportion of variance accounted for by either factor was between 24 and 39% (*R*^2^ predictor, *p* < 0.01). In this study, stronger negative correlations were observed between binaural fusion range and VGRM for both NH listeners and bilateral HA users, especially in the co-located target-masker configuration. As reported in Table 2, the proportion of variance accounted for by binaural pitch fusion for VGRM was 50% (*R*^2^ predictor, *p* = 0.014) for NH listeners, and 72% (*R*^2^ predictor, *p* < 0.001) for bilateral HA users, which are higher than the amount of variance explained by age (*R*^2^ = 0.07, *p* = 0.23 in the current study; *R*^2^ = 0.02, *p* < 0.52 in Glyde et al., 2013) or hearing loss (*R*^2^ = 0.51, *p* < 0.01 in the current study; *R*^2^ = 0.39, *p* < 0.001 in Glyde et al., 2013) alone. Hence, broad binaural fusion could be a stronger predictor for reduced VGRM than age or hearing loss. It should be noted that the significance of this proportion of variance was observed only in the co-located target-maskers spatial configuration. We also confirmed that significance of the correlation was eliminated when binaural cues were not provided (i.e., at two monaural listening conditions; see Table 2), indicating that the correlation is not explained by poorer frequency discrimination or other factors that might also lead to broad binaural fusion. In particular, some subjects with broad fusion had larger VGRM under monaural listening compared to binaural listening, consistent with an interpretation of binaural interference arising from broad binaural fusion.

Regarding the relationship of sound localization acuity to SRM, a negative correlation was observed, but was not statistically significant. As reported in Table 3, the proportion of variance accounted for by localization acuity for SRM was low at 25% (*R*^2^ predictor, *p* = 0.115) for NH listeners and 22% (*R*^2^ predictor, *p* = 0.123) for bilateral HA users. A similar finding was also reported in the study by Srinivasan et al. (2021) with 22% of variance (*R*^2^ predictor, *p* = 0.033) accounted for NH listeners. The lack of statistical significance in this study is likely due to the small sample size for each listener group, along with the small effect size. There is likely to be an effect of localization acuity, but this effect seems to be small. One reason for the small effect size is that localization acuity with multiple sounds from multiple sound sources may differ from that for a single sound, especially when there is broad binaural fusion. In such cases, fusion of multiple sounds from different spatial locations may occur, leading to an illusion of a single sound source with a diffuse spatial percept, and thus poor localization acuity. Thus, a better predictor of ability to benefit from SRM may be localization ability of more than one sound source presented simultaneously. It should also be noted that the current study estimated the absolute localization acuity without considering front-back confusion in the subject’s responses. In this study, three NH and four HA subjects showed some degree of front-back confusion rates in their absolute localization acuity measurements, especially for the two narrowband signal conditions. The application of a more rigorous angular analysis, perhaps one in which front-back errors are considered, should be explored in future studies.

Interestingly, the multiple regression analysis results (Table 4) showed that the pure-tone average was a strong predictor for the variations of all outcomes measured in this study: (1) the masking release (>18% as *R*^2^ predictor, *p* < 0.045); (2) the binaural pitch fusion range (29% as *R*^2^ predictor, *p* = 0.009); and (3) the absolute localization acuity at three different stimuli (>35% as *R*^2^ predictor, *p* < 0.003). However, age couldn’t predict those variations (*p* > 0.227). These results indicate that the degree of hearing loss itself could be a common factor to explain degraded binaural sensitivity involved in speech-on-speech masking performance and related to pitch and spatial perception. In addition, although the correlation between age and degree of hearing loss was not found in the current study (*r* = 0.078, *p* = 0.724), it is well known that the age of the listeners is often allowed to covary with hearing loss. Furthermore, as mentioned in the introduction, the reduce binaural sensitivity could be caused by a reduction in higher-order processing such as cognitive and linguistic abilities (Besser et al., 2015). Therefore, future work will need to involve listeners who vary widely in age regardless of hearing status to separately examine the effects of age and hearing loss as factors.

In conclusion, this is the first study to demonstrate an important role of abnormally broad binaural pitch fusion in reduced binaural benefits for speech perception in multi-talker listening environments for both NH and HI listeners. The findings demonstrate that masking release from both voice gender and spatial cues is much smaller for HA users than NH listeners, and that the reduced benefit from voice gender cues is explained by abnormally broad binaural pitch fusion. Thus, for HI listeners, it will be critically important to help restore sharply tuned pitch fusion across ears for optimal binaural benefit in noise environments, especially when benefit from spatial cues is limited. Increased understanding of factors that affect binaural benefits for speech perception for HI listeners is clinically essential for the future design of training- and device-based rehabilitative strategies to improve speech perception in quiet and noise.

## Data availability statement

The raw data supporting the conclusions of this article will be made available by the authors, without undue reservation.

## Ethics statement

The studies involving human participants were reviewed and approved by the Institutional Review Boards (IRBs) of both Oregon Health and Sciences University and the Portland VA Medical Center. The patients/participants provided their written informed consent to participate in this study.

## Author contributions

YO, FG, and LR designed the experiments. YO, CH, NS, and AD performed the experiments. YO analyzed the data. All authors contributed to the article, discussed the results at all states, and approved the submitted version.

## References

[B1] Albogast T. L. Mason C. R. Kidd G. Jr. (2002). The effect of spatial separation on informational and energetic masking of speech. *J. Acoust. Soc. Am.* 112 2086–2098. 10.1121/1.1510141 12430820

[B2] Allen K. Charlile S. Alais D. (2008). Contributions of talker characteristics and spatial location to auditory streaming. *J. Acoust. Soc. Am.* 123 1562–1570. 10.1121/1.2831774 18345844

[B3] Baltzell L. S. Swaminathan J. Cho A. Y. Lavandier M. Best V. (2020). Binaural sensitivity and release from speech-on-speech masking in listeners with and without hearing loss. *J. Acoust. Soc. Am.* 147 1546–1561. 10.1121/10.0000812 32237845 PMC7060089

[B4] Bernstein J. G. W. Goupell M. J. Schuchman G. I. Rivera A. L. Brungart D. S. (2016). Having two ears facilitates the perceptual separation of concurrent talkers for bilateral and single-side deaf cochlear implantees. *Ear Hear.* 37 289–302. 10.1097/AUD.0000000000000284 26886027 PMC4869863

[B5] Besser J. Festen J. M. Goverts T. Kramer S. E. Pichora-Fuller M. K. (2015). Speech-in-speech listening on the LiSN-S test by older adults with good audiograms depends on cognition and hearing acuity at high frequencies. *Ear Hear.* 36 24–41. 10.1097/AUD.0000000000000096 25207850

[B6] Best V. Marrone N. Mason C. Kidd G. Jr. (2012). The influence of non-spatial factors on measures of spatial release from masking. *J. Acoust. Soc. Am.* 131 3103–3110. 10.1121/1.3693656 22501083 PMC3339507

[B7] Best V. Mason C. R. Kidd G. Jr. (2011). Spatial release from masking in normally hearing and hearing-impaired listeners as a function of the temporal overlap of competing Talkers. *J. Acoust. Soc. Am.* 129 1616–1625. 10.1121/1.3533733 21428524 PMC3078033

[B8] Bolia R. S. Nelson W. T. Ericson M. A. Simpson B. D. (2000). A speech corpus for multitalker communications research. *J. Acoust. Soc. Am.* 107 1065–1066. 10.1121/1.428288 10687719

[B9] Brown A. D. Rodriquez F. A. Portnuff C. D. F. Goupell M. J. Tollin D. J. (2016). Time-varying distortions of binaural information by bilateral hearing aids: Effects of nonlinear frequency compression. *Trends Hear.* 20 1–15. 10.1177/2331216516668303 27698258 PMC5051674

[B10] Brungart D. S. (2001). Informational and energetic masking effects in the perception of two simultaneous talkers. *J. Acoust. Soc. Am.* 109 1101–1109. 10.1121/1.1345696 11303924

[B11] Brungart D. S. Chang P. S. Simpson B. D. Wang D. (2009). Multitalker speech perception with ideal time-frequency segregation: Effects of voice characteristics and number of talkers. *J. Acoust. Soc. Am.* 125 4006–4022. 10.1121/1.3117686 19507982

[B12] Byrne D. Noble W. (1998). Optimizing sound localization with hearing aids. *Trends Amplif.* 3 51–73. 10.1177/108471389800300202 25425879 PMC4172152

[B13] Cherry E. C. (1953). Some experiments on the recognition of speech, with one and with two ears. *J. Acoust. Soc. Am.* 25 975–979. 10.1121/1.1907229

[B14] Ching T. Y. van Wanrooy E. Dillong H. (2007). Binaural-bimodal fitting or bilateral implantation for managing severe to profound deafness: A review. *Trends Amplif.* 11 161–192. 10.1177/1084713807304357 17709573 PMC4111363

[B15] Dai L. Best V. Shinn-Cunningham B. G. (2018). Sensorineural hearing loss degrades behavioral and physiological measures of human spatial selective auditory attention. *Proc. Natl. Acad. Sci. U.S.A.* 115 E3286–E3295. 10.1073/pnas.1721226115 29555752 PMC5889663

[B16] Darwin C. J. Brungart D. S. Simpson B. D. (2003). Effects of fundamental frequency and vocal-tract length changes on attention to one of two simultaneous talkers. *J. Acoust. Soc. Am.* 114 2913–2922. 10.1121/1.1616924 14650025

[B17] Ellinger R. L. Jakien K. M. Gallun F. J. (2017). The role of interaural differences on speech intelligibility in complex multi-talker environments. *J. Acoust. Soc. Am.* 141 EL170–EL176. 10.1121/1.4976113 28253635 PMC5392079

[B18] Ericson M. A. Brungart D. S. Simpson B. D. (2004). Factors that influence intelligibility in multitalker speech displays. *Int. J. Aviat. Psychol.* 14 313–334. 10.1207/s15327108ijap1403_6 25127327

[B19] Flanagan J. L. (1965). *Speech analysis, synthesis and perception.* New York, NY: Springer-Verlag, 176–184. 10.1007/978-3-662-00849-2

[B20] Florentine M. Popper A. N. Fay R. R. (2011). “Chapter 2,” in *Loudness*, eds Marks L. E. Florentine M. (New York, NY: Springer), 17–56.

[B21] Folstein M. F. Folstein S. E. McHugh P. R. (1975). “Mini-mental state”: A practical method for grading the cognitive state of patients for the clinician. *J. Psychiatr. Res.* 12 189–198. 10.1016/0022-3956(75)90026-6 1202204

[B22] Füllgrabe C. Moore B. C. Stone M. A. (2015). Age-group differences in speech identification despite matched audiometrically normal hearing: Contributions from auditory temporal processing and cognition. *Front. Aging Neurosci.* 6:347. 10.3389/fnagi.2014.00347 25628563 PMC4292733

[B23] Gallun F. J. Diedesch A. C. (2013). Exploring the factors predictive of informational masking in a speech recognition task. *Proc. Meet. Acoust.* 19:060145. 10.1121/1.4799107

[B24] Gallun F. J. Kampel S. D. Diedesch A. C. Jakien K. M. (2013). Independent impacts of age and hearing loss on spatial release in a complex auditory environment. *Front. Neurosci.* 7:252. 10.3389/fnins.2013.00252 24391535 PMC3870327

[B25] Gallun F. J. Mason C. R. Kidd G. (2005). Binaural release from informational masking in a speech identification task. *J. Acoust. Soc. Am.* 118 1614–1625. 10.1121/1.1984876 16240821

[B26] Gaudrain E. Başkent D. (2018). Discrimination of voice pitch and vocal-track length in cochlear implant users. *Ear Hear.* 39 226–237. 10.1097/AUD.0000000000000480 28799983 PMC5839701

[B27] Gifford R. H. Dorman M. F. Skarzynski H. Lorens A. Polak M. Driscoll C. L. W. (2013). Cochlear implantation with hearing preservation yields significant benefit for speech recognition in complex listening environments. *Ear Hear.* 34 413–425. 10.1097/AUD.0b013e31827e8163 23446225 PMC3742689

[B28] Gifford R. H. Grantham D. W. Sheffield S. W. Davis T. J. Dwyer R. Dorman M. F. (2014). Localization and interaural time difference (ITD) thresholds for cochlear implant recipients with preserved acoustic hearing in the implanted ear. *Hear. Res.* 312 28–37. 10.1016/j.heares.2014.02.007 24607490 PMC4036440

[B29] Glyde H. Cameron S. Dillon H. Hickson L. Seeto M. (2013). The effects of hearing impairment and aging on spatial processing. *Ear Hear.* 34 15–28. 10.1097/AUD.0b013e3182617f94 22941406

[B30] Henry K. S. Heinz M. G. (2013). Effects of sensorineural hearing loss on temporal coding of narrowband and broadband signals in the auditory periphery. *Hear Res.* 303 39–47. 10.1016/j.heares.2013.01.014 23376018 PMC3688697

[B31] Kidd G. Mason C. R. Rohtla T. L. Deliwala P. S. (1998). Release from masking due to spatial separation of sources in the identification of nonspeech auditory patterns. *J. Acoust. Soc. Am.* 104 422–431. 10.1121/1.423246 9670534

[B32] Levitt H. (1971). Transformed up-down methods in psychoacoustics. *J. Acoust. Soc. Am.* 49 467–477. 10.1121/1.1912375 5541744

[B33] Litovsky R. Y. (2012). Spatial release from masking. *Acoust. Today* 8 18–25. 10.1121/1.4729575

[B34] Litovsky R. Y. Johnstone P. M. Godar S. Agrawal S. Parkinson A. Peters R. (2006). Bilateral cochlear implants in children: Localization acuity measured with minimum audible angle. *Ear Hear.* 27 43–59. 10.1097/01.aud.0000194515.28023.4b 16446564 PMC2651156

[B35] Lorenzi C. Gatehouse S. Lever C. (1999). Sound localization in noise in normal-hearing listeners. *J. Acoust. Soc. Am.* 105 1810–1820. 10.1121/1.426719 10089604

[B36] Marrone N. Mason C. R. Kidd G. (2008). Evaluating the benefit of hearing aids in solving the cocktail party problem. *Trends Amplif.* 12 300–315. 10.1177/1084713808325880 19010794 PMC2836772

[B37] Misurelli S. M. Litovsky R. Y. (2012). Spatial release from masking in children with normal hearing and with bilateral cochlear implants: Effect of interferer asymmetry. *J. Acoust. Soc. Am.* 132 380–391. 10.1121/1.4725760 22779485 PMC3407161

[B38] Misurelli S. M. Litovsky R. Y. (2015). Spatial release from masking in children with bilateral cochlear implants and with normal hearing: Effect of target-interferer similarity. *J. Acoust. Soc. Am.* 138 319–331. 10.1121/1.4922777 26233032 PMC4506300

[B39] Moulines E. Laroche J. (1995). Non-parametric techniques for pitch-scale modification of speech. *Speech Commun.* 16 175–205. 10.1016/0167-6393(94)00054-E

[B40] Oh Y. Reiss L. (2017a). Voice gender release from masking in cochlear implant users is correlated with binaural pitch fusion. *J. Acoust. Soc. Am.* 141:3816. 10.1121/1.4988444

[B41] Oh Y. Reiss L. A. (2017b). Binaural pitch fusion: Pitch averaging and dominance in hearing-impaired listeners with broad fusion. *J. Acoust. Soc. Am.* 142 780–791. 10.1121/1.4997190 28863555 PMC5648564

[B42] Oh Y. Reiss L. A. (2020). Binaural pitch fusion: Binaural pitch averaging in cochlear implant users with broad binaural fusion. *Ear Hear.* 41 1450–1460. 10.1097/AUD.0000000000000866 33136622 PMC7501189

[B43] Oh Y. Bridges S. E. Schoenfeld H. Layne A. O. Eddins D. (2021). Interaction between voice-gender difference and spatial separation in release from masking in multi-talker listening environments”. *JASA Express Lett.* 1:084404. 10.1121/10.0005831 34713273 PMC8547139

[B44] Oh Y. Srinivasan N. K. Hartling C. L. Gallun F. J. Reiss L. A. J. (2022). Differential effects of binaural pitch fusion range on the benefits of voice gender differences in a “cocktail party” environment for bimodal and bilateral cochlear implant users. *Ear Hear.* Available online at: https://journals.lww.com/ear-hearing/Abstract/9900/Differential_Effects_of_Binaural_Pitch_Fusion.70.aspx 10.1097/AUD.0000000000001283 PMC9957805 36395512

[B45] Reiss L. A. Eggleston J. L. Walker E. P. Oh Y. (2016). Two ears are not always better than one: Mandatory vowel fusion across spectrally mismatched ears in hearing-impaired listeners. *J. Assoc. Res. Otolaryngol.* 17 341–356. 10.1007/s10162-016-0570-z 27220769 PMC4940290

[B46] Reiss L. A. Fowler J. R. Hartling C. L. Oh Y. (2018a). Binaural pitch fusion in bilateral cochlear implant users. *Ear Hear.* 39 390–397. 10.1097/AUD.0000000000000497 28945657 PMC5821581

[B47] Reiss L. A. Ito R. A. Eggleston J. L. Wozny D. R. (2014). Abnormal binaural spectral integration in cochlear implant users. *J. Assoc. Res. Otolaryngol.* 15 235–248. 10.1007/s10162-013-0434-8 24464088 PMC3946135

[B48] Reiss L. A. Molis M. R. (2021). An alternative explanation for difficulties with speech in background talkers: Abnormal fusion of vowels across fundamental frequency and ears. *J. Assoc. Res. Otolaryngol.* 22, 443–461. 10.1007/s10162-021-00790-7 33877470 PMC8329143

[B49] Reiss L. A. Molis M. Simmons S. Katrina L. (2018b). Effects of broad binaural fusion and hearing loss on dichotic concurrent vowel identification. *J. Acoust. Soc. Am.* 143:1942. 10.1121/1.5036358

[B50] Reiss L. A. Shayman C. S. Walker E. P. Bennett K. O. Fowler J. R. Hartling C. L. (2017). Binaural pitch fusion: Comparison of normal-hearing and hearing-impaired listeners. *J. Acoust. Soc. Am.* 143 1909–1920. 10.1121/1.4978009 28372056 PMC5848869

[B51] Richter M. E. Dillon M. T. Buss E. Leibold L. J. (2021). Sex-mismatch benefit for speech-in-speech recognition by pediatric and adult cochlear implant users. *JASA Express Lett.* 1:084403. 10.1121/10.0005806 PMC8340498 34396366

[B52] Shaw E. A. G. (1974). Transformation of sound pressure level from the free field to the eardrum in the horizontal plane. *J. Acoust. Soc. Am.* 56 1848–1861. 10.1121/1.1903522 4443484

[B53] Souza P. E. Boike K. T. Witherall K. Tremblay K. (2007). Prediction of speech recognition from audibility in older listeners with hearing loss: Effects of age, amplification, and background noise. *J. Am. Acad. Audiol.* 18 54–65. 10.3766/jaaa.18.1.5 17252958

[B54] Srinivasan N. K. Jakien K. M. Gallun F. J. (2016). Release from masking for small separations: Effects of age and hearing loss. *J. Acoust. Soc. Am.* 140 EL73–EL78. 10.1121/1.4954386 27475216 PMC5392088

[B55] Srinivasan N. K. Staudenmeier A. Clark K. (2021). Effect of gap detection threshold and localisation acuity on spatial release from masking in older adults. *Int. J. Audiol.* 18 1–8. 34793273 10.1080/14992027.2021.1961168

[B56] Swaminathan J. Mason C. R. Streeter T. Best V. Roverud E. Kidd G. (2016). Role of binaural temporal fine structure and envelope cues in cocktail-party listening. *J. Neurosci.* 36 8250–8257. 10.1523/JNEUROSCI.4421-15.2016 27488643 PMC4971368

[B57] Visram A. S. Kulk K. McKay C. M. (2012). Voice gender differences and separation of simultaneous talkers in cochlear implant users with residual hearing. *J. Acoust. Soc. Am.* 132 EL135–EL141. 10.1121/1.4737137 22894312

[B58] Wiggins I. M. Seeber B. U. (2013). Linking dynamic-range compression across the ears can improve speech intelligibility in spatially separated noise. *J. Acoust. Soc. Am.* 133 1004–1016. 10.1121/1.4773862 23363117

[B59] Yost W. A. (2017). Spatial release from masking based on binaural processing for up to six maskers. *J. Acoust. Soc. Am.* 141, 2093–2106. 10.1121/1.4978614 28372135 PMC5848840

